# Items

**Published:** 1888-01

**Authors:** 


### ITEMS.

### How Scarlet Fever Comes to Michigan.

The Michigan State Board of Health has received information from Dr. Sifton, health officer of Sutton's Bay township, which illustrates, in a striking way, how this country gets contagious diseases from the old countries. October 2, 1887, a family arrived in Sutton's Bay, Leelanaw county, direct from Norway. The family came over in the S. S. Ohio, of the Inman line, reaching New York September 30. Scarlet fever was on board the steamer during the passage, one child dying before the landing, and "several more were sick in the same way." One child of this family was taken sick with scarlet fever the day after reaching New York. The family, however, proceeded over the New York Central and the Lake Shore & Michigan Southern to Michigan; then over the Detroit, Grand Haven & Milwaukee, and the Grand Rapids & Indiana to Traverse City; then to Sutton's Bay. Another child of the family has since come down with the disease. The family had a certificate, signed by the surgeon of the steamer, that they had been protected by vaccination against small-pox; so they passed without detention the quarantine authorities at the port of New York, after they had been exposed to a contagious disease which causes more deaths by far in this country than small-pox causes.

---

### A Decision as to Costs in Suits for Malpractice.

For a considerable time a suit has been pending against a physician of Boston, founded on a charge of malpractice involving some operative procedure for removal of the ovaries. It has been tried four times. The first three trials resulted in disagreement of the juries. The fourth trial recently terminated in a verdict for the defendant. But the item of most interest to members of our profession is, that at the opening of the last trial the counsel for the defense applied for and obtained a decision of the court, that all the costs of the four trials should be paid by the plaintiff, in case the final verdict of the jury should be in favor of the defendant. The example thus furnished in this trial is worthy of the careful consideration of all judges before whom charges of malpractice are brought, a large proportion of which have no other foundation than a desire to extort money from the defendant sufficient to secure a good fee for the prosecuting counsel.—*Journal of The American Medical Association.*

---

### Diabetes Mellitus.

Professor C. W. Purdy says: "In the medicinal treatment of diabetes mellitus, if the disease be of true nervous origin, preference is given to bromide of arsenic in the form of Gilliford's solution, which may be given in doses of from five to ten drops three times daily in water, after food. The average dose necessary to influence favorably the disease has been found to range at about eight or nine drops. If, at the end of about three weeks, sugar be still present in the urine, the dose should be increased to ten drops."

The above solution should not be confounded with Clemens' solution of arsenic bromide, as the dose of the latter ranges much smaller—two to five drops.

---

The health officers of Baltimore blame the imperfect quarantine at New York for an outbreak of diphtheria in the Italian quarter of that city. It will be well if the country next summer does not have to place the responsibility for an outbreak of cholera in the same quarter.—*Chicago Evening Journal.*

